# Mixed Infection Phylacogen in the Treatment of Diabetic Gangrene

**Published:** 1914-08

**Authors:** V. A. Pchellas

**Affiliations:** Buffalo, N. Y.


### Mixed Infection Phylacogen in the Treatment of Diabetic Gangrene.

By V. A. PCHELLAS, M. D., BUFFALO, N. Y.

It is a well-known fact in the practice of medicine that infectious conditions of all kinds are peculiarly fatal when the patient is coincidently afflicted with diabetes. A person with disturbed carbohydrate metabolism is so prone to severe bacterial infection that, when a physician meets with a fulminating case in which extreme bacterial activity is manifested, he at once examines the urine for the presence of sugar.

Of the many conditions to which the diabetic patient is liable, perhaps none may become of such great import as the spontaneous gangrene of an extremity and the infection that always accompanies it. Therapeutic measures are in many instances useless and repeated amputations prove to be but a temporizing measure. For that reason anything that offers a hope of eradicating the infection and prolonging the life of the patient will be welcome in the treatment of this serious affection.

Mixed Infection Phylacogen has been employed by the medical profession a sufficient length of time so that most physicians are more or less familiar with it. Being made up of metabolic products of a variety of micro-organism, it is peculiarly effective against different infectious conditions in which a single type of organism cannot be said to be predominant.

In regard to the case that I wish to report, I fully realize that a single instance does not fully establish the therapeutic value of a preparation in the treatment of a given disease; nevertheless it should encourage physicians to administer the product in other cases so that the end results may be carefully studied and the exact status of the remedy accurately determined. This patient was sixty years of age, and because of this and the fact that the blood and urine were heavily loaded with sugar makes it indeed noteworthy that any therapeutic agent could so stimulate the forces of immunity as to successfully eradicate the infectious condition.

Case A. Mrs. B., age 60.

Diagnosis: Diabetic gangrene.

History: Past history negative except for the presence of diabetes mellitis. On Christmas day patient noticed a small sore on the finger. The member became swollen and exceedingly painful and in a few days the end of the finger became gangrenous. The swelling had extended until it involved half the hand. January 1st, 1912, the finger had become gangrenous past the second joint with no pain, but the hand was swollen to the wrist. There was abundant sugar in the urine, with no temperature present. January 2, the finger was amputated at the third joint, but the swelling in the hand continued to extend and the purulent discharge increased.

| | | | | | |
|---|---|---|---|---|---|
| January | 10, | Mixed | Infection | Phylacogen, | 2 Cc. subcutaneously |
| " | 11, | " | " | " | 2 Cc. " |
| " | 12, | " | " | " | 2 Cc. " |
| " | 15, | " | " | " | 2 Cc. " |
| " | 16, | " | " | " | 2 Cc. " |
| " | 18, | " | " | " | 2 Cc. " |
| " | 20, | " | " | " | 2 Cc. " |
| " | 22, | " | " | " | 2 Cc. " |
| " | 26, | " | " | " | 5 Cc. " |
| " | 28, | " | " | " | 5 Cc. " |
| " | 30, | " | " | " | 5 Cc. " |

At the time the discharge was much less and there was marked general improvement. After each injection the swelling decreased to return the next day but the redness and swelling gradually grew less.

When the Phylacogen was suspended the swelling would increase, but grew less after the next dose. Further doses were given as follows:

| | | | | | |
|---|---|---|---|---|---|
| February, | 1, | Mixed | Infection | Phylacogen, | 5 Cc. subcutaneously |
| " | 3, | " | " | " | 5 Cc. " |
| " | 5, | " | " | " | 5 Cc. " |
| " | 7, | " | " | " | 5 Cc. " |
| " | 9, | " | " | " | 5 Cc. " |
| " | 12, | " | " | " | 5 Cc. " |
| " | 15, | " | " | " | 5 Cc. " |
| " | 18, | " | " | " | 5 Cc. " |
| " | 21, | " | " | " | 5 Cc. " |

The patient was then discharged, cured.

Judging from this case alone it would seem that Phylacogen exerts an influence over the vital protective forces of the body peculiar to itself. Especially is this true as it is evidently capable of exerting that action even when the body tissues are saturated with sugar.

I wish to take this opportunity to briefly mention two cases of furunculosis successfully treated with this preparation, the results being obtained so promptly as to be truly remarkable.

Case B. Mr. A. D., age 20.

Diagnosis: Furunculosis.

History: A profuse crop of boils appeared on the patient's neck. January 25, 1912. 5 Cc. of Mixed Infection Phylacogen were administered subcutaneously with the result that the boils promptly dried up and disappeared.

Case C. Mr. W. N., age 21.

Diagnosis: Furunculosis.

History: January 15, 1912, patient developed boils on the arm, which increased in number until the entire member was covered and several appeared on the other arm. I gave calcium sulphide without effect. January 20, pleurisy developed with cough, profuse expectoration, and night sweats.

January 24, 5 Cc. of Mixed Infection Phylacogen were administered subcutaneously. This dose was repeated on January 27.

The boils glazed over, dried up, and disappeared. The pleurisy, cough, and night sweats quickly cleared up and the patient rapidly gained in weight. He was discharged, to all intents and purposes, perfectly well.

While these latter cases were not complicated by diabetes, yet they serve to demonstrate the effectiveness of Mixed Infection Phylacogen when directed against suppurating processes in general. So true is this that a physician or surgeon is entirely justified in resorting to the preparation in the treatment of these conditions, and especially is it advisable for the latter to administer it to prevent or control infectious processes in the many traumatic conditions that he is called upon to treat.

1481 E. Genesee St.

